# Dr. Eber S. Carlisle

**Published:** 1909-01

**Authors:** 


Dr. Eber S. Carlisle, of Los Angeles, Cal., died at his home in that city, December 8, 1908, aged 65 years. He graduated at the University of Buffalo in 1868.

